# A Systematic Review of Genomic Regions and Candidate Genes Underlying Behavioral Traits in Farmed Mammals and Their Link with Human Disorders

**DOI:** 10.3390/ani11030715

**Published:** 2021-03-06

**Authors:** Amanda B. Alvarenga, Hinayah R. Oliveira, Shi-Yi Chen, Stephen P. Miller, Jeremy N. Marchant-Forde, Lais Grigoletto, Luiz F. Brito

**Affiliations:** 1 Department of Animal Sciences, Purdue University, West Lafayette, IN 47907, USA; alvarena@purdue.edu (A.B.A.); hrojasde@purdue.edu (H.R.O.); chen3505@purdue.edu (S.-Y.C.); laisgrigo@hotmail.com (L.G.); 2 Centre for Genetic Improvement of Livestock, Department of Animal Biosciences, University of Guelph, Guelph, ON N1G 2W1, Canada; 3 Farm Animal Genetic Resources Exploration and Innovation Key Laboratory of Sichuan Province, Sichuan Agricultural University, Chengdu 625014, China; 4 American Angus Association, Saint Joseph, MI 64506, USA; SMiller@angus.org; 5 Livestock Behavior Research Unit, United States Department of Agriculture—Agricultural Research Service (USDA–ARS), West Lafayette, IN 47907, USA; Jeremy.marchant-forde@usda.gov; 6 Department of Veterinary Medicine, College of Animal Science and Food Engineering, University of Sao Paulo, Pirassununga 05508, São Paulo, Brazil

**Keywords:** animal welfare, behavioral genetics, cattle, domestication, livestock behavior, pigs, sheep, temperament, workability

## Abstract

**Simple Summary:**

This study is a comprehensive review of genomic regions associated with animal behavior in farmed mammals (beef and dairy cattle, pigs, and sheep) which contributes to a better understanding of the biological mechanisms influencing the target indicator trait and to gene expression studies by suggesting genes likely controlling the trait, and it will be useful in optimizing genomic predictions of breeding values incorporating biological information. Behavioral mechanisms are complex traits, genetically controlled by multiple genes spread across the whole genome. The majority of the genes identified in cattle, pigs, and sheep in association with a plethora of behavioral measurements (e.g., temperament, terrain use, milking speed, tail biting, and sucking reflex) are likely controlling stimuli reception (e.g., olfactory), internal recognition of stimuli (e.g., neuroactive ligand–receptor interaction), and body response to a stimulus (e.g., blood pressure, fatty acidy metabolism, hormone signaling, and inflammatory pathways). Six genes were commonly identified between cattle and pigs. About half of the genes for behavior identified in farmed mammals were also identified in humans for behavioral, mental, and neuronal disorders. Our findings indicate that the majority of the genes identified are likely controlling animal behavioral outcomes because their biological functions as well as potentially differing allele frequencies between two breed groups (subjectively) clustered based on their temperament characteristics.

**Abstract:**

The main objectives of this study were to perform a systematic review of genomic regions associated with various behavioral traits in the main farmed mammals and identify key candidate genes and potential causal mutations by contrasting the frequency of polymorphisms in cattle breeds with divergent behavioral traits (based on a subjective clustering approach). A total of 687 (cattle), 1391 (pigs), and 148 (sheep) genomic regions associated with 37 (cattle), 55 (pigs), and 22 (sheep) behavioral traits were identified in the literature. In total, 383, 317, and 15 genes overlap with genomic regions identified for cattle, pigs, and sheep, respectively. Six common genes (e.g., *NR3C2*, *PITPNM3*, *RERG*, *SPNS3*, *U6*, and *ZFAT*) were found for cattle and pigs. A combined gene-set of 634 human genes was produced through identified homologous genes. A total of 313 out of 634 genes have previously been associated with behavioral, mental, and neurologic disorders (e.g., anxiety and schizophrenia) in humans. Additionally, a total of 491 candidate genes had at least one statistically significant polymorphism (*p*-value < 0.05). Out of those, 110 genes were defined as having polymorphic regions differing in greater than 50% of exon regions. Therefore, conserved genomic regions controlling behavior were found across farmed mammal species and humans.

## 1. Introduction

Livestock species have been selected for specific behavioral responses since domestication, which occurred approximately 10,000 years ago [1,2,3,4]. This has been done to facilitate human–animal interactions and farming practices, especially in intensive production systems incorporating a multitude of technological equipment [3,5,6]. Studies have suggested the relationship between animal behavior and handler/farmer safety, overall welfare of the animals, workability, and production efficiency (e.g., average daily gain, carcass yield, longevity, milk yield, reproductive performance, and udder health [7,8,9,10,11,12,13,14,15,16,17,18,19,20,21,22]). Animal-handling activities are the third leading cause of morbidity in farms, causing from 12% to 24% of farm injuries [23]. Along with handler/farmer wellness, animal wellbeing is an overarching theme [24,25]. Principles of animal welfare are, for example, related to good human–animal relationships and animals’ positive reactions when exposed to novel objects [26].

A large number of indicators of altered behavior and welfare have been proposed over time, including physiological variables (e.g., cortisol levels [27,28,29]) and activities (e.g., communication, aggression and social structures, biological rhythms and sleep, reproductive behavior, maternal behavior, development behavior, learning, feeding behavior, and miscellaneous behavioral disorders [30,31,32,33,34,35,36,37,38,39,40,41,42,43,44,45,46,47]). In beef and dairy cattle, the four most common behavioral indicators are flight speed (i.e., time spent for an animal to exit the chute [33,48,49]), terrain use (i.e., the animal ability to explore the terrain or new environment [34,50]), temperament score (e.g., from 1—docile animal, to 5—aggressive animal; [37,48,51,52,53,54,55,56,57]), and milking speed [37,38,54,55,58,59,60,61,62,63,64]. Traits such as infanticide, feeding behavior, and tail biting in pigs [32,47,65,66,67,68,69], and vocalization in sheep [35,70] are commonly assessed behavioral traits. In addition to the aforementioned traits, sucking reflex is a measurement of newborn mammal behavior [71]. The calf sucking reflex pattern interferes with colostrum intake and whole-milk feeding duration, which is directly associated with overall animal welfare and nursery workability [71]. Nonetheless, those commonly measured behavioral traits are difficult to measure due to a multitude of factors interfering with the measurements, such as the evaluator (i.e., handler/farmer responsible for the scoring), environmental conditions, single/few behavioral records in the animal’s lifetime, labor time, and handling. The recent development of cost-effective high-throughput phenotyping tools has the potential to revolutionize selective breeding for behavior and welfare. These tools include activity monitors (e.g., pedometers, and microchip sensors [72]), video-imagining [73], and physiological biomarkers [28] (see Brito et al. [74] for a review). 

Animal behavior is a complex phenotype often assessed by a series of measurements as aforementioned, which depends on genetic characteristics of each individual as well as various environmental factors that they are subjected to. Most behavioral traits are heritable, with heritability estimates ranging from 0.02 to 0.69 [30,33,35,37,38,39,40,41,42,43,45,46,47,50,55,56,60,63,71,75]. Selection for altered behavioral mechanisms has been very effective and generated breeds or populations with different coping strategies to different stressor factors, advocating that genetic progress can be achieved [53,56,75]. Therefore, functional genomic analysis can play an essential role in better understanding the genetic architecture of behavior and the biological and physiological pathways involved in the expression of each trait.

The majority of genomic studies investigating the genetic background of behavioral traits has been performed within a breed, usually under the same environmental conditions and recorded for a single or few behavior indicators [35,45,48]. However, there is evidence that some genomic regions controlling behavioral traits are conserved across breeds and species [56]. The animal behavioral responses are also shaped by multi-environmental factors, such as different rearing conditions, production system (e.g., free-ranging, confinement livestock farming), geographic variations (e.g., resulting in gut microbiome differences, which is related to the conundrum of behavior-microbe interactions [76]), and policy/culture makers (e.g., bull fighting breeds in Spain [77]). Gathering information from different studies can render robust knowledge regarding animal behavior because it may capture a multitude of environmental factors [78]. In addition, this learning provides new insights on breeds and species lacking in erudition. The main objectives of this study were to (1) systematically review genomic regions associated with behavioral traits in beef and dairy cattle, pigs, and sheep; (2) perform within and across species functional annotation of candidate genes; (3) identify important genomic regions conserved across livestock species; and (4) evaluate the differences in allele frequency of polymorphisms in cattle breeds with divergent behavioral characteristics based on independent whole-genome sequence data as a complementary analysis. The breeds were clustered in two groups in a subjective manner as no formal comparison of temperament characteristics across all breeds has been reported in the literature. No behavioral studies at the genomic level were found for goats.

## 2. Material and Methods

### 2.1. Data Gathering and Editing

A search for genomic regions associated with behavioral traits in cattle, pigs, goats, and sheep was conducted using the Animal QTL database (Animal QTLdb; www.animalgenome.org/cgi-bin/QTLdb/index; accessed date: 1 February 2020), National Center for Biotechnology Information (PubMed; www.ncbi.nlm.nih.gov/pubmed/; accessed date: 1 February 2020), and Google Scholar (https://scholar.google.com; accessed date: 2 January 2020) web search engines. The search queries consisted of combinations of keywords based on the following criteria: species term (e.g., bovine, cattle, caprine, goat, swine, pig, ovine, sheep); type of analysis [e.g., candidate genes, genome-wide association, gene expression, genome-wide association studies (GWAS), single-step GWAS (ssGWAS)]; and trait-related term (e.g., aggression, behavior, chute score, courtship, cortisol, dominant, docility, exit velocity, flight, fearfulness, grazing, infanticide, learning ability, locomotion, lesion score, pen score, savaging, stress, social, sociability, struggle, speed, sucking behavior, temperament, tameness, time spent, tail biting, welfare). A file compiling all genomic regions associated with any previously defined species and behavior-related traits was downloaded from the Animal QTLdb (www.animalgenome.org/cgi-bin/QTLdb/index; accessed date: 1 February 2020). Various keyword combinations were searched in PubMed and Google Scholar, such as “(‘bovine’ AND ‘GWAS’ AND ‘behavior’) OR (‘cattle’ AND ‘GWAS’ AND ‘behavior’)”. All genomic regions reported in peer-reviewed scientific papers, conference proceeding papers, and theses from the three web search engines were summarized. The genomic regions reported as candidate in association with the target traits were catalogued and, thus, no statistical test was provided in all the studies. Furthermore, the relevance criterion for genomic regions varied across studies (e.g., Kramer et al. [55] selected genomic regions with largest effects while Hanna et al. [53] used a *p*-value criterium). Ideally, a meta-analysis using the summary statistics for all markers included in all the studies should be performed. However, this information was not available. We recommend that future studies report the summary statistics for all SNP markers and not only the significant regions. Beforehand, no behavioral studies at the genomic level were found for goats.

**Figure 1:**
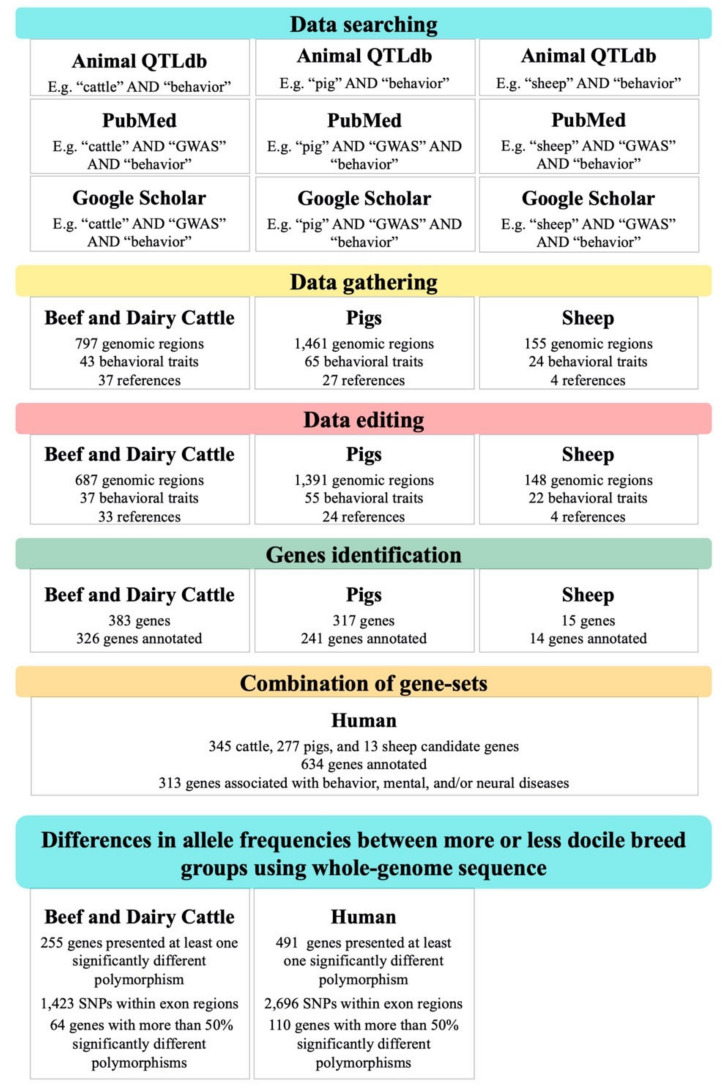
Workflow of the systematic review of genomic regions associated with behavior-related traits in farmed mammals and human homologous genes.

After gathering all genomic regions associated with behavioral traits, a quality control procedure was performed. The first control consisted of removing redundant studies published by the same author set and using the same dataset and methodologies. The priority order was peer-reviewed paper, thesis, and conference proceeding papers (peer-reviewed, but not yet reported as a full paper). Subsequently, genomic regions were retained if there was at least one of the following information: labelled genomic region (i.e., gene name; gene Ensembl identification; single nucleotide polymorphism identification; microsatellite identification that renders a genome coordinate in base pair (bp) unit), or genomic region annotated in physical map (i.e., in bp). Lastly, genomic regions with the difference between start and end chromosome position lower than one mega base pair (Mbp; 1 Mbp = 1,000,000 bp) remained for further analyses. The systematic review scheme is presented in Figure 1. Please note that no studies were found for goats (i.e., thus not included in the illustrative scheme).

### 2.2. Identification of Candidate Genes

Candidate genes within the target genomic regions were identified by either marker names or genome coordinate (in base pairs) filters using the *biomaRt* R package [79,80]. In this step, the reference genome uploaded was the same used in the original publication. To guarantee direct comparisons, the physical positions of all candidate genes were converted to uniform genome coordinates based on the latest reference genome available in the *biomaRt* R package [79,80] for cattle (ARS-UCD1.2 [81]), pigs (Sscrofa11.1 [82]), and sheep (OAR 3.0 [83]).

### 2.3. Common Genomic Regions

The overlapping genomic regions within and across species were evaluated based on gene matching (i.e., how many times the gene repeated within species, or how many times the gene was repeated across species datasets). In other words, candidate genes were annotated for each genomic information at a time. The goal of this first step was to link the gene information (e.g., gene name, gene Ensembl identification, genomic coordinates) with the publication, trait, and originally identified genomic region. Subsequently, the number of times that the gene was repeated represents the gene overlapping within species and across pairs of species (i.e., cattle versus pigs, cattle versus sheep, and pigs versus sheep). In order to identify overlapping genes across species, homologous genes in cattle, pigs, and sheep were obtained using the *biomaRt* R package [79,80].

### 2.4. Functional Analyses

#### 2.4.1. Functional Analyses within Species

To reveal the functional implications of the detected candidate genes, an enrichment analysis was performed in terms of biological processes (BP), cellular components (CC), and molecular functions (MF) of Gene Ontology terms [84] and metabolic pathways of the Kyoto Encyclopedia of Genes and Genomes (KEGG; [85]) in the DAVID database [86] for each species. The significance of the terms enriched was tested by Bonferroni multiple-test correction, as implemented in the DAVID platform [86]. As a complementary analysis, an independent whole-genome sequence dataset for various cattle breeds [87] was used to detect the exonic polymorphisms (single nucleotide polymorphisms (SNPs)) for all candidate genes identified in cattle. We focused on exonic SNPs because their variations are most likely to have functional implications and they are the most complete datasets available in the referred data source [87]. There is still a lack of studies in the literature comparing behavioral responses across breeds, especially across animals reared under similar management conditions. Therefore, the breeds were categorized into two groups with likely differential temperament characteristics based on the literature (e.g., [88]), breed association and extension web reports, and personal communication with livestock producers and extension specialists. The first group (Group 1) is classified as more temperamental and includes six breeds, namely Brahman, Charolais, Gyr (or Gir), Nellore (or Nelore), Piedmontese, and Shorthorn Zebu, while the second group (Group 2) consisted of Hereford, Holstein, Jersey, Angus, and Red Angus, with more docile characteristics [87]. The animals and breeds available had an average DNA sequencing coverage of equal to 12.12 (Group 1; from 4.9 to 18.6) and 13.6 (Group 2; from 6.67 to 24.35). Both coding regions and untranslated regions (UTRs) were targeted for SNP detection within the candidate genes among 45 animals in Group 1 (i.e., 9 Brahman, 14 Charolais, 3 Gyr, 4 Nellore, 5 Piedmontese, and 10 Shorthorn Zebu) and 119 animals in Group 2 (i.e., 21 Hereford, 45 Holstein, 12 Jersey, 25 Angus, and 16 Red Angus) [87]. This grouping approach is partially subjective and could influence the results, but there is enough evidence about the differences in temperament across breeds in the literature. Future studies should also include additional breeds as well as larger and more balanced datasets. In total, there were 11,035 SNPs tested across autosomal chromosomes. All SNP with minor allele frequency (MAF) lower than 5% in Group 1 were subjected to statistical testing for intergroup differences using Fisher’s exact test (false discovery rate adjusted *p*-value ≤ 0.05). In order to minimize bias due to *Bos taurus taurus* and *Bos taurus indicus* differences, an adjustment for SNPs significantly different between both subspecies breeds ((Brahman, Nelore, and Shorthorn Zebu) versus (Charolais, and Piedmontese)) were performed. By way of explanation, we have removed all SNP with the MAF lower than 5% in the *Bos taurus taurus* groups that were statistically different from the MAF in *Bos taurus indicus* group. Finally, the statistically significant SNPs adjusted to *Bos taurus taurus* and *Bos taurus indicus* differences (*p*-value ≤ 0.05; null hypothesis: allele frequencies of Group 1 and Group 2 are equal) were classified as potential causal mutations. Candidate genes with a ratio of significantly different SNPs and all SNPs within the gene region higher than 50% suggest strong association with animal behavior. Statistical power of the Fisher’s exact test was evaluated using the *power.fisher.test* function from the *statmod* package in R software [81,89] considering a significance level of 0.05 and 1000 population simulation. The average of the statistical power across loci was 52%. SNP frequency comparisons were only applied in the cattle gene-set because there were insufficient sequenced-breed data publicly available for pigs and sheep. It is worth highlighting that this is a complementary analysis and limited by the sample size and number of breeds from each cattle subspecies. However, the results obtained by contrasting allele frequencies between more or less docile breed groups provide additional support for the systematic review findings.

#### 2.4.2. Functional Analysis across Species

A functional analysis across species was based on a combined candidate gene-set of all three species. Accordingly, the human orthologs were retrieved for all genes across species. The final gene-set was a merger of the three species gene-set by human homologous identification. As an example, the genes ENSBTAG00000027182 and ENSSSCG00000037766 identified in association with behavior in cattle and pigs, respectively, are equivalent to ENSG00000151623 Ensembl gene identification in humans.

A functional annotation was performed using DAVID [86] based on the human database. Additionally, human disease annotations were performed using the dataset available in the DisGeNET (www.disgenet.org [90]; accessed date: 7 June 2020) database. The gene-disease association was retrieved from DisGeNET according to the animal model databases (mainly mouse and rat data) and considering three disease classes: behavior and behavior mechanisms, mental disorders, and nervous system diseases. Additionally, a causal mutation gene–disease annotation was also retrieved from DisGeNET in order to highlight key genes that have evidence of actual causal associations with behavioral traits in humans or model animals. In this sense, based on all data sources available in DisGenNET (i.e., information from UniProt, PsyGeNET, Orphanet, the CGI, CTD, ClinGen, the Genomics England PanelApp, RGD, MGD, CTD, the Human Phenotype Ontology, Clinvar, the GWAS catalog, GWASdb, LHGDN and BeFree; please see the complete description at www.disgenet.org [90]; accessed date: 20 December 2020), the search queries were “causal mutation” and the three aforementioned disease classes (i.e., behavioral mechanisms, mental disorders, and nervous system diseases). The genome coordinates of human genes were annotated based on the latest reference genome available in the *biomaRt* R package (i.e., genome assembly GRCh38.p13 [79,80,91]).

Finally, we have tested the genetic divergence of two cattle breed groups with contrasting temperament characteristics based on genes annotated in other farmed mammal species. We have retrieved the cattle homologous identification for all genes identified in pigs and sheep and tested differences in allele frequency as conducted in Section 2.4.1. In total, 13,529 SNPs located on autosome chromosomes and within target genic regions (genes identified in cattle, pigs, and sheep) were tested. The dataset and procedure are described in Section 2.4.1.

## 3. Results

A total of 797, 1461, and 155 genomic regions associated with behavioral traits for cattle (*Bos taurus*), pigs (*Sus scrofa*), and sheep (*Ovis aries*), respectively, were compiled in this study. The studies gathered were from animals raised across many countries (Figure S1). In total, 20 breeds and their crosses in cattle, eight breeds and their crosses in pigs, and five breeds in sheep were evaluated in genomic behavior studies. The breeds represented in this systematic review are presented in Table 1.

### 3.1. Beef and Dairy Cattle

**Figure 2:**
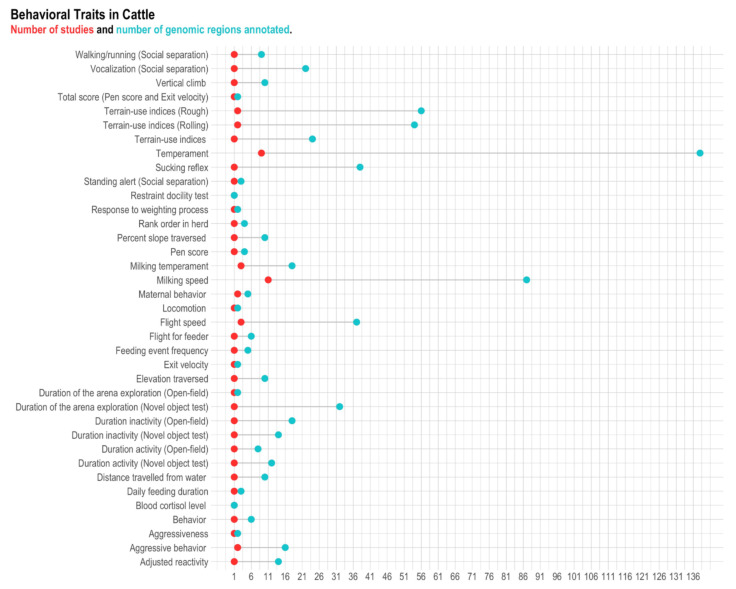
Number of genomic regions retrieved and references for each behavior-related trait in cattle. Red dots represent the number of references, and blue dots represent the number of genomic regions. Traits with a single-colored dot (e.g., restraint docility test) mean that the number of studies is equal to the number of genomic regions (therefore, the dots overlapped).

**Figure 3:**
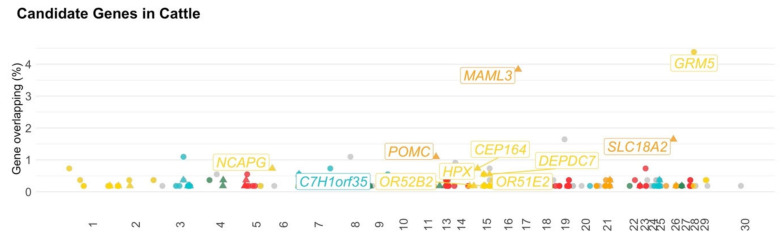
Candidate genes associated with behavior-related traits in cattle. The x-axis is gene chromosome position; y-axis represents the gene overlapping expressed in percentage (i.e., ratio of times of gene appearance and total number of genomic regions); colored dots represent genes with at least one significantly different polymorphism between Groups 1 and 2; triangle dots represent the genes with the highest proportion of significant polymorphisms out of all the polymorphisms (Group 1 versus Group 2); different colors represent the chromosomes; labels are the gene symbols for (i) genes with the highest proportion of significant polymorphisms out of all the polymorphisms while contrasting Group 1 and Group 2, and (ii) genes reported in relevant pathways in association with the target traits; chromosome 30 represents the X (sex) chromosome.

A total of 797 genomic regions located across all chromosomes (i.e., 29 autosomal and X chromosomes) were found to be associated with behavioral traits in cattle (Table S1A). After the quality control, 687 genomic regions remained for further analyses, and those regions were associated with 37 traits (Figure 2 and Table 2). The remaining genomic regions were distributed across 29 autosomal and X chromosomes and harbored 383 genes, in which 326 were annotated genes (Table S2A and Figure 3).

A total of seven genes were identified to be associated with behavior in more than one study (Table S3A). The highest overlapping on either within- and across-studies (i.e., same genes identified across genomic regions within a publication and across different publications) was found on BTA29 (between 7,007,877 and 7,197,732 bp), which contains the *GRM5* gene (glutamate metabotropic receptor-5) [34,50]. The *GRM5* gene was associated with terrain-use indices across studies [34,50]. The second genomic region that overlapped the most among studies was found on BTA17 (from 17,836,577 to 18,018,432 bp), which is associated with the *MAML3* gene (mastermind-like transcriptional coactivator-3) and also linked with terrain-use indices [34,50]. Other common genomic regions among studies were located on BTA3 (from 89,165,719 to 89,209,186 bp; *C8B* gene—complement C8 beta chain, associated with temperament [52,53]), BTA8 (from 59,723,669 to 59,785,504 bp; *RUSC2* gene—RUN and SH3 domain containing-2, associated with terrain-use indices [34,50]), BTA11 (from 74,117,511 to 74,124,989 bp; *POMC* gene—proopiomelanocortin, associated with temperament and pen score [48,92]), BTA21 (from 47,398,496 to 47,734,138 bp; *MIPOL1* gene—mirror-image polydactyly-1, associated with temperament and distance travelled from water [50,53]), and BTA26 (from 37,562,768 to 37,602,774 bp; *SLC18A2* gene—solute carrier family-18 member-A2). The *SLC18A2* gene has been associated with temperament, pen score, and milking speed behavior indicator traits [48,54].

The functional annotation using 383 cattle candidate genes did not render significant terms after the Bonferroni multiple-test correction. However, biologically important terms related to behavior responses were identified, such as neuronal action potential, regulation of blood pressure, and steroid hormone-mediated signaling biological processes. Various metabolic pathways associated with behavior identified in this study are aldosterone regulation, olfaction, sensory transduction, glutamatergic synapse, and vasopressin-regulated water reabsorption (Table S4). The GO terms for biological processes, molecular functions, cellular components, and metabolic pathways are shown in Table S4.

To identify potential causal mutations within candidate genes associated with behavioral traits in cattle, the allele frequencies of SNPs located in exon regions for each gene were compared between more temperamental breeds (Group 1) and more docile breeds (Group 2). Using a whole-genome sequence database, 292 genes were identified containing 6098 SNPs within exon regions. After adjustments for subspecies bias *(Bos taurus taurus* versus *Bos taurus indicus*) and false discovery rate (FDR), 1423 SNPs located across 255 genes significantly different in allele frequencies between the docile and temperamental cattle groups. The significant genes are represented by colored dots in Figure 3, and all genes with their respective SNPs, *p*-values, and statistical power are presented in Table S5A. Furthermore, 64 genes had a ratio of significant to non-significant SNPs greater than 50 percent. These genes with higher proportion of significant SNPs are represented by triangle dots in Figure 3. Additionally, 106 genes had statistical differences between their allele frequencies between more docile and more temperamental breed groups considering a power of Fisher’s exact test higher than 80% (Table S5B).

### 3.2. Pigs

**Figure 4:**
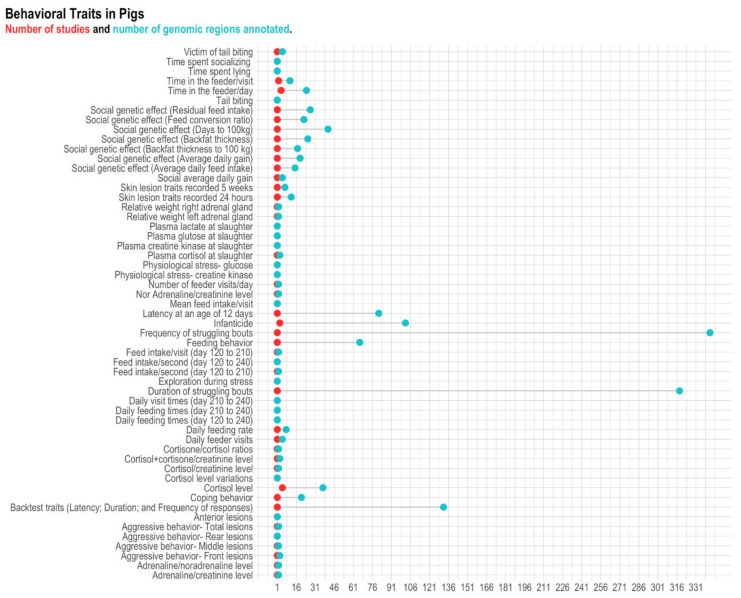
Number of genomic regions and reference per behavior-related trait in pigs. The red dots represent the number of references (studies), and blue dots represent the number of genomic regions. Traits with a single-colored dot (e.g., time spent socializing) mean that the number of studies is equal to the number of genomic regions (therefore, the dots overlapped)**.**

**Figure 5:**
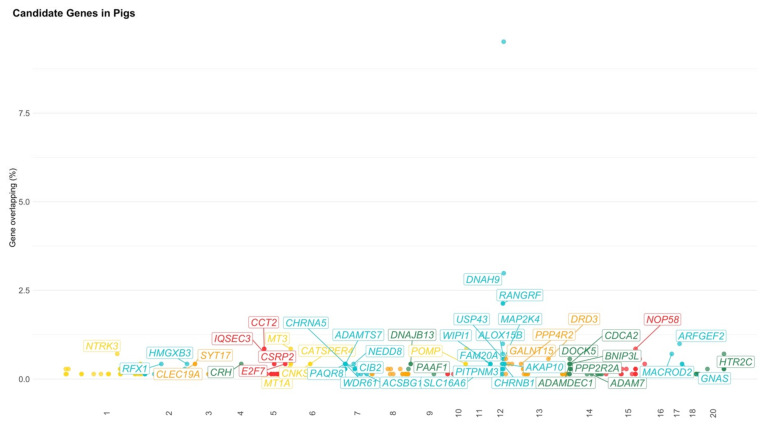
Manhattan plot of candidate genes in pigs. The x-axis represents the chromosomes; y-axis represents the gene overlapping in percentage (i.e., ratio of times of gene appearance and total number of genomic regions); the color represents the chromosomes; labelled boxes represent annotated genes with number of appearances higher than 3; chromosome 20 is the X (sex) chromosome.

A total of 1461 genomic regions located on SSC1-18 and SSCX chromosomes were found to be associated with behavior indicators in pigs (Table S1B). After the data editing, 1391 genomic regions associated with 55 different behavioral traits remained for further analyses (Figure 4 and Table 3). In total, 317 positional genes were found on 18 autosome chromosomes (i.e., SSC1-SSC18) and on the X chromosome (SSCX), in which 241 were annotated genes (Figure 5 and Table S2B).

Six genes were identified in more than one reference, such as *CSE1L* (chromosome segregation-1 like, located on SSC17; associated with frequency of struggling bouts and social genetic effect for average daily feed intake [45,46]), *MACROD2* (mono-ADP ribosylhydrolase-2, located on SSC17; associated with duration and frequency of struggling bouts and daily feeding rate [46,68]), *NR3C2* (nuclear receptor subfamily-3 group-C member-2, located on SSC8; associated with social genetic effect and cortisol+cortisone/creatinine level [45,99]), and *PLCB1* (phospholipase C beta-1, located on SSC17; associated with skin lesion traits and infanticide [32,100]). The genes with the highest recurrence among genomic regions were located on SSC12 (from 55,757,851 to 56,021,816 bp; 11% of the total number of genomic regions), which is an unknown gene (i.e., ENSSSCG00000024467; associated with duration and frequency of struggling bouts, backtest traits, and latency [46]). The genes and their recurrence are shown in Figure 5 and Table S3B.

No biological or metabolic pathways were significant after the Bonferroni multiple-test correction (*p*-value < 0.05). However, the main biological processes with important implications in behavior responses were related to synaptic transmission (dopaminergic), ubiquitin-dependent protein catabolic process, long-term synaptic potentiation, long-term synaptic depression, negative regulation of microtubule polymerization, and locomotion. Additionally, the main metabolic pathways linked to behavioral traits were dopaminergic synapse, neurotrophin signaling pathways, GnRH, hippo signaling pathway, insulin secretion, and melanogenesis. The GO terms for biological processes, molecular functions, cellular components, and KEGG are presented in Table S6.

### 3.3. Sheep

**Figure 6:**
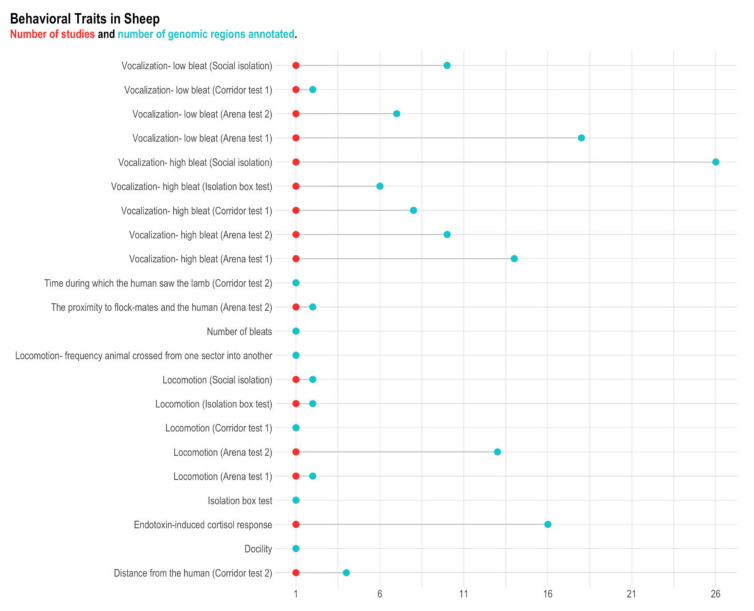
Number of genomic regions annotated and reference per behavior-related trait in sheep. The red dots represent the number of references, and the blue dots represent the number of genomic regions. Traits with a single-colored dot (e.g., time during which the human saw the lamb) mean that the number of references is equal to the number of genomic regions.

**Figure 7:**
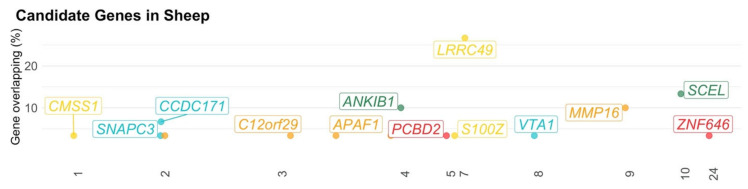
Manhattan plot of candidate genes in sheep. The x-axis represents the chromosome; y-axis represents the gene overlapping in percentage (i.e., ratio of times of gene appearance and total number of genomic regions); the color represents the chromosomes; labelled boxes represent the symbol for all annotated genes.

A total of 155 genomic regions located in 13 chromosomes (i.e., OAR1-10, OAR15, OAR22, and OAR24) were found to be associated with behavior indicators in sheep (Table S1C). After the quality control, 148 genomic regions associated with 22 behavior indicators remained for further analyses (Figure 6 and Table 4). These genomic regions contained 15 positional genes, of which 14 are annotated genes in the latest reference genome available in the *biomaRt* R package (OAR 3.0; Figure 7 and Table S2C). No overlapping genes were observed among studies. The overlapping genes within studies among genomic regions are shown in Figure 7 and Table S3C. Due to the small number of positional genes identified for sheep, no GO and KEGG terms were enriched.

### 3.4. Common Regions across Farmed Mammals

The 383, 317, and 15 genes identified for cattle, pigs, and sheep, respectively, were merged through the identification of human homologous genes. Few genes reported in these farmed species had no human homologous genes. Therefore, the combined gene-set in the human genome was comprised of 634 genes, 345 of which came from cattle, 277 from pigs, and 13 from sheep (note that there was one human homologous gene that overlapped between cattle and pigs, out of six commonly identified genes for these species).

Six common genes were found between cattle (383 genes) and pig gene-sets (317 genes). The common genes were *NR3C2* (nuclear receptor subfamily-3 group-C member-2), *PITPNM3* (PITPNM family member-3), *RERG* (RAS like estrogen regulated growth Inhibitor), *SPNS3* (sphingolipid transporter-3), *U6* (U6 spliceosomal RNA), and *ZFAT* (zinc finger and AT-hook domain containing; Table S7). The nuclear receptor subfamily-3 group-C member-2 gene (*NR3C2)* is located on BTA17 and SSC8, and it was associated with temperament in cattle and social genetic effect (days to 100 kg) and cortisol+cortisone/creatinine levels in pigs. Both *PITPNM3* and *SPNS3* genes are located on BTA19 and SSC12, and they were associated with temperament in cattle and struggling bouts (frequency and duration) and latency at an age of 12 days in pigs. The RAS like estrogen regulated growth inhibitor gene *(RERG)* is located on chromosome 5 in both cattle and pigs, and it was associated with milking temperament in cattle and feeding behavior in pigs (Tables S3A and S3B). The U6 spliceosomal RNA gene (*U6*; located on BTA3 and SSC6) was associated with temperament, maternal behavior, and sucking reflex in cattle, and feeding behavior and adrenaline/creatinine level in pigs (Tables S3A and S3B). Finally, *ZFAT* is located on BTA14 and SSC4, being mainly associated with milking speed and frequency of struggling bouts in cattle and pigs, respectively (Tables S3A and S3B). No common genes were found for cattle and sheep or pigs and sheep. A subset of genes is presented in Table 5, which were selected based on the recurrence within and across species and if it was reported in the literature as biologically relevant for behavioral traits.

In addition to the functional enrichment, human disease annotation was performed using the combined gene-set. In total, 313 genes were associated with 315 human diseases classified as behavioral and mental disorders and nervous system diseases (Table S8). The main diseases were anxiety, autism, depression, attention deficit hyperactivity disorder, antisocial personality disorder, intellectual disability, and schizophrenia. Those diseases were mainly associated with two genes of the *ADAM* family (i.e., *ADAM10* and *ADAMTS2*), *CDC6, DEPDC7*, two genes of the *FAM* family (i.e., *FAM135B* and *FAM177A1*), *HPX, MAOA*, *MAOB*, *MAML3*, *NR3C2, POMC, PLCB,* and eight genes of the solute carrier family (e.g., *SLC13A5*, *SLC18A2*, *SLC25A4*, *SLC5A2*, and *SLC6A2*). The gene–human disease associations are presented in Table S8. Furthermore, 61 genes are from causal mutation analysis, which provides additional evidence of gene–disease association (Table S9).

Dopaminergic synapse metabolic pathway was the only significant term after Bonferroni multiple-test correction, which was characterized by 15 genes, including *DRD3* (dopamine receptor-D3), *GNAS* (GNAS complex locus), *MAOB* (monoamine oxidase B), *MAPK8* (mitogen-activated protein kinase-8), *PPP2R5D* (protein phosphatase-2 regulatory subunit B’delta), *PPP2R2A* (protein phosphatase-2 regulatory subunit B’alpha), *PRKCB* (protein kinase C-beta), *PLCB1* (phospholipase-C beta-1), *SCN1A* (sodium voltage-gated channel alpha subunit-1), and *SLC18A2* (solute carrier family-18 member-A2; Table S7). However, other behavior-related terms were also rendered from the candidate genes, such as processes involved in sensory perception, central nervous system, and metabolic processes outside of the central nervous system. Pathways, cellular components, and molecular functions that might be modulated by sensory perception, stimuli induction (e.g., sensory receptors), and/or internal recognition of the stimuli were olfaction, sensory transduction, olfactory transduction, detection of chemical stimulus involved in sensory perception, vision, and response to stimulus (Table S7). Thirteen genes from the olfactory receptor family (e.g., *OR2B6*, *OR11H1*, *OR51E2*, *OR56A1*, *OR52L1*, *OR9Q2*, *OR52J3*, *OR52B2*, and *OR11H12*) were identified in the present study in association with temperament traits in cattle populations [53] (Tables S3A and S7).

Other biologically relevant functions related to behavior, but that were non-significant, were metalloprotease, neuroactive ligand–receptor interaction, dilated cardiomyopathy, and hormone signaling pathway (e.g., GnRH, aldosterone, oxytocin, estrogen, glucagon, and dopamine hormones) (Table S7). Important genes such as *NTRK3* (neurotrophic receptor tyrosine kinase-3) and *DRD3* (dopamine receptor D3) were presented in those biologically important pathways. The terms related to biological and metabolic processes outside of the central nervous system were salivary secretion, hormone signaling pathway (e.g., insulin and thyroid hormones), growth factor, fatty acid metabolism, regulation of blood pressure, inflammatory pathways, and locomotion (Table S7). Candidate genes well known for playing a role in productive traits, e.g., meat quality, were also identified in this study including *MUSK* (muscle associated receptor tyrosine kinase, associated with feeding behavior in pigs [47]) and *CAST* (calpastatin, associated with sucking reflex in cattle [71]).

The evaluation of allele frequency divergence between two contrasting temperament groups in cattle population were also performed for genes identified in other mammalian species (i.e., pigs and sheep). Out of the 634 genes, 556 were identified containing 11,929 SNPs within exon regions. After the adjustments for *Bos taurus taurus* versus *Bos taurus indicus* differences and FDR, 2,696 SNPs located in 491 genes significantly different in allele frequencies between the more docile and more temperamental cattle breed groups. The genes with their respective SNPs, *p*-values, and power of Fisher’s exact test are presented in Table S10A. In total, 110 genes presented higher proportion of significantly different polymorphisms (62 genes from cattle, 44 genes from pigs, and 4 from sheep). Additionally, 219 genes presented statistical difference between their allele frequencies across more docile and more temperamental breeds considering a power of Fisher’s exact test higher than 80% (Table S10B). Those genes play important roles in biologically important pathways associated with behavior, such as dopaminergic mechanisms, insulin secretion, GnRH, signal transduction, hippo signaling, and learning or memory.

## 4. Discussion

Animal behavior can be assessed through a plethora of measurements (as reviewed by Brito et al. [74]). Similar performance and outcomes for behavioral traits in cattle (i.e., docility, temperament score, and qualitative behavior assessments through four-platform standing scale; [113]) and in pigs (i.e., struggling bouts and feeding behavior; [114]) were observed. However, null genetic correlations were also found among other behavior-related traits, such as activity score (i.e., mild and excited scoring), struggling bouts, and feeding behavior [114]. The diversity of behavioral assessments and environmental conditions might interfere with the outcomes of studies assessing the genetic background of the traits (e.g., genetic variation and association of genomic regions) [115]. Therefore, a study compiling genomic variations across populations and environmental conditions represents a robust understanding of the genetic landscape of behavioral traits. Examples of some contributions of this study are (i) the potential inclusion of genomic regions in genotyping platforms or next generation sequencing panels to explore genetic diversity and genomic prediction of breeding values from a behavioral perspective, and (ii) prioritization of genes and gene-associated variants in animal behavior analyses in species with poor elucidation of the genetic background for the specific indicator trait.

This study focused on farmed mammals, such as beef and dairy cattle, pigs, and sheep. Although there are studies investigating the genetic background of behavioral traits in other mammalian livestock species (e.g., dairy goats’ grazing behavior [116] and milking temperament [117]), none of them included genomic information. Chickens are another livestock species that are just as cognitively, emotionally, and socially complex as mammals [118]. Studies associating genomic and behavioral outcomes have also been performed [e.g., [119]], but they were not included and further discussed in this study because we aimed to focus on more genetically related species. A large number of genomic regions and candidate genes affecting the expression of behavioral traits in beef and dairy cattle, pigs, and sheep have been published over the past decades. In this study, a total of 68 publications (37 in cattle, 27 in pigs, and four in sheep) with data collected in more than 19 countries (16 in cattle, 10 in pigs, and three in sheep) reported more than 2400 genomic regions (797 in cattle, 1461 in pigs, and 155 in sheep) associated with 131 behavioral traits (43 cattle, 64 pigs, and 24 sheep) (Table S1). The discussion presented below was conducted considering the species jointly, i.e., cattle, pigs, sheep, and combined gene dataset.

There is evidence of conserved genes and genomic regions across various species, especially livestock species and humans [120]. This includes studies reporting translational genomic regions controlling behavioral traits in cattle and humans [56], pigs and humans [105], as well as chicken and humans [119]. For instance, MacLeod et al. [56] reported candidate genes (i.e., *NCOA7*, *GAD2*, *PDGFD*, *TMPRSS5*, *DRD2*, *IQSEC1*, *MAOB*, *PTPRF*, *SLC25A16*, *TMCO5A*, and *SNRPB2*) associated with dairy cattle temperament that were previously linked to human neuropsychiatric disorders (e.g., schizophrenia). In our study, about half of the candidate genes for livestock behavior (313/634) were found to be associated with 315 mental, behavioral, and neurologic disorders in humans, including anxiety, autism, attention deficit hyperactivity disorder, antisocial personality disorder, bipolar and mental disorders, borderline personality disorder, depression, eating disorder, intellectual disability, stress, and schizophrenia (Table S8). Furthermore, 61 genes were reported in causal association analysis, which provides additional evidence of gene-behavioral traits association (Table S9). Those translational genes controlling both neuronal and psychologic human diseases and agricultural behavioral traits likely retain a common biological mechanism. This similarity of biological mechanisms across mammalian species represents a great opportunity to translate knowledge generated across disciplines. For example, there is a much larger number of studies linking psychological and behavioral variables in humans to genomic regions. Thus, homologous genes identified in humans might provide important information for livestock studies, including the use of such information in biology-driven genomic selection.

In addition to the translational genes between farmed species and humans, six candidate genes were shared between cattle and pigs in this study (i.e., *NR3C2, RERG*, *PITPNM3*, *SPNS3*, *U6*, and *ZFAT*). Two of these genes (i.e., *NR3C2* and *RERG*) were associated with neuropsychiatric human disorders (Table S8). Additionally, five out of those six common genes presented significant polymorphism within their exonic regions by comparing the two groups differing in temperament (Group 1—more temperamental, and Group 2—more docile; Table S10A; i.e., *NR3C2*, *RERG*, *PITPNM3*, *SPNS3*, and *ZFAT*), and two presented a power of Fisher’s exact test higher than 80% (i.e., *PITPNM3* and *ZFAT;* Table S10B). *PITPNM family member-3* (*PITPNM3*) and *ZFAT* genes were previously reported in association with vision (i.e., phototransduction [121]) and immune system (i.e., B and T lymphocytes [122]) in humans, respectively. Furthermore, *ZFAT* was also identified as a candidate gene for semi-lethality in Aberdeen Angus [123]. Despite the fact that these genes have not been directly associated with behavioral traits, animal behavior may be indirectly affected by immune function (i.e., through stress-hormone level alterations; [124]), and phototransduction has inherent sensory implications (e.g., stimuli reception).

Some important candidate genes associated with behavioral traits were located on the sex chromosomes. The genes included *MAOB* identified in cattle (i.e., associated with temperament; Table S3A), and *OBP*, *HTR2C*, *MIR1912*, *U6*, ENSSSCG00000012257 (unknown gene in pigs, but the homolog name in other species is *MAOA*), ENSSSCG00000012125 (unknown gene in pigs, but the homolog name in other species is *OFD1*) identified in pigs (e.g., associated with feeding behavior and adrenaline/creatinine level; Table S3B). Three out of seven genes (i.e., *HTR2C*, *MAOA*, and *OFD1*) were previously associated with behavioral disorders in humans, such as aggressive behavior, antisocial personality, and low frustration tolerance (Table S8). Two (*MAOA* and *OFD1*) were also reported as causal mutations affecting behavioral, mental, and neuronal disorders (Table S9). Additionally, in farmed mammals, the protein produced by *OFD1* gene is localized in the centrosome and the basal body of primary cilia [125,126], which have crucial roles in cell signaling pathways and cellular homeostasis [127,128]. A behavioral response can be concisely described as a stimulus inducing the central nervous system to initiate an appropriate response via signaling [129].

Other genes located on the sex chromosomes biologically associated with behavior were *MAOB* (i.e., monoamine oxidase B, identified in cattle) and *OBP* (identified in pigs). Monoamine oxidase B gene (*MAOB*) was associated with the mood modulation through phenylethylamine (PEA) metabolism in mice [130]. Additionally, *MAOB*-deficient mice experienced an increased reactivity to stress [130]. Finally, proteins of the *OBP* gene mediate the reception of signals for biological fluids and organs as a passive transporter for hydrophobic odorant molecules (lipids, steroid hormones, and retinoids) [131]. Accordingly, the *OBP* gene might influence pheromone mechanisms related to species- or breed-specific signaling, social hierarchy establishment, nursing behavior [132], and changes in feeding behavior [131]. Therefore, there is strong evidence of sex chromosomes modulating behavior responses, suggesting the importance of including polymorphisms located on the sex chromosome in genomic analyses.

Animal reaction is mainly controlled by complex neural networks, which results in physiological alterations. Many pathways associated with synaptic transmission and neuroreceptor were found in our study. A specific term involved in neural networks and brain function is apoptosis. Apoptosis is one of the controlling mechanisms of the neural pool and synaptic matching between neurons during the development of the nervous system [133]. Ho-Shing and Dulac [133] have described genes affecting the brain processes in mice (i.e., neural-apoptosis), in which *KCNK9* gene was included. Potassium two pore domain channel subfamily-K member-9 gene (*KCNK9*) plays a role in mediating neuronal excitability [134], promoting apoptosis in granule cells [135], and encoding a protein that affects aldosterone secretion [136]. Interesting, the *KCNK9* gene is a causal mutation in humans associated with Birk-Barel mental retardation dysmorphism syndrome, a rare maternally inherited disease (Table S9 and [137]).

One of the domestication theories observes the reduction of neural crest-derived tissues due to selection for behavior [4]. Neural crest cells are stem cells that first appear during the early embryogenesis stage at the neural tube, eventually migrating ventrally throughout the body in both the cranium and the trunk [4]. In this context, the post-embryonic development term was identified in our study, which includes the *SLC18A2* gene. Specifically, *SLC18A2* (associated in cattle; gene from solute carrier family) plays a role in chemical synaptic transmission, locomotive behavior, and dopaminergic and serotonergic synapses. In total, 14 genes from the solute carrier family were annotated in this study (seven in cattle associated with temperament, pen score, milking speed, and rank order in herd [48,53,54,55]; seven in pigs associated with feeding behavior, frequency of struggling bouts, and social genetic effect [45,46,47]), and they were reported in behavior-related pathways, such as long-term synaptic potentiation and depression. Out of those genes from *SCL* family, 12 genes (12/14) and 4 genes (including *SLC18A2*) were statistically and causally associated, respectively, with human behavioral disorders. Furthermore, five genes presented a power of Fisher’s exact test for the allele frequency between more docile and more temperamental breed groups higher than 80% (Table S10B). Therefore, similar to observed in humans for neurodegenerative disorders [138], we suggest that genes from the solute carrier family might play an important role in behavioral traits.

Behavioral responses are induced by external or internal stimuli (e.g., animal interaction, human handling, temperature alterations, and transportation). Pathways related to olfactory mechanisms were identified (Tables S4, S6, and S7). Genes from olfactory receptor family (*OR*; e.g., *OR2B6* and *OR51E2*) were predominantly reported in those terms. Olfactory pathways were related to receptors and sensory transduction. Unknown individuals introduced to pigs stimulate new olfactory pathways, oftentimes contributing to aggressive behavior [139]. Olfactory signaling is a cue used for social recognition [139] in addition to evoking reproductive behaviors in mammalian livestock species [140,141].

Another example of physiological alteration in response to behavior is blood pressure. Two neuropeptides out of many factors affecting the blood pressure are oxytocin and arginine vasopressin, which are well known for their roles in reproduction and homeostatic processes [142]. These molecules were also associated with the animal defensive behavior (social learning and behavior) [142,143]. In humans, genes encoding oxytocin and arginine vasopressin pathways have been associated with individual variation in social recognition, social attachment phenotypes, parental behavior, and psychiatric phenotypes such as autism [143]. Oxytocin and blood pressure pathways were represented in our study (Table S7). Thereafter, oxytocin levels can be related to behavioral reactivity, thereby implicating this molecule as a potential alternative biomarker of animal behavior. Oxytocin can be measured through blood (not optimal because restraining the animals would cause stress by itself), saliva [144], and milk samples.

Finally, several studies have reported significant phenotypic and genetic correlation between economically important traits and animal behavior, such as reproductive traits (e.g., pregnancy rate [145]), meat quality (e.g., tenderness, taste [7,8,12,146]), and welfare. Some mechanisms associated with genetic correlation are pleiotropy, linkage disequilibrium between trait-specific QTLs, and/or linkage disequilibrium between QTL and markers [147]. Known genes and pathways reportedly playing a role in the expression of other productive traits that have been identified in this study are the *CAST* gene and sexual hormone pathways. Gonadal steroids can differentially affect the hypothalamic–pituitary–adrenal system [148], which is crucial to behavior. For instance, it can prepare the animal for a threatening stimulus (e.g., fight-or-flight) [4].

The *CAST* gene (calpastatin) was systematically reviewed in this study (Table S2A), having association with the bovine sucking reflex [71]. The *CAST* gene plays a key role in postmortem tenderization of meat and has been proposed to be involved in muscle protein degradation of living tissue [149]. Interesting, the frequency of the favorable allele for *CAST* gene is different between breeds with contrasting meat quality and temperament characteristics. In Angus populations, the average frequency of the favorable A allele for *CAST* is about 0.80 [150], while in the Nellore breed, it is 0.56 [151]. The Angus breed is well known for tender meat and tame behavior compared to Nellore, which has problems with tenderness and assortative behavior. Extending purebreds to crossbreds, Angus and Nellore crosses are generally known by animals with good temperament as well as good meat quality, being also associated at the genetic level with a higher frequency of the *CAST* favorable allele (frequency of *A* equal to 0.90; [151]).

Beta-adrenergic signaling regulates the calpastatin levels in cattle, which affect beef tenderness and, consequently, meat quality [7]. Some pathways involving beta-adrenergic receptors and/or presenting similar biological functions (e.g., dopaminergic, glutamatergic, and serotonergic synapses) were also found in this study. In parallel with their behavioral control, the neuromodulators (i.e., dopamine, glutamate, and serotonin) of these processes are well known for their importance in neural development and function [152], stress response [153], sexual behavior [154], and compulsive disorder [155].

## 5. Conclusions

In order to gain a better biological insight into behavioral genomics in livestock, the present study systematically reviewed multi-breed and multi-species candidate genomic regions controlling farmed mammal behavior. The majority of annotated candidate genes had divergent allele frequencies between contrasting temperament cattle breeds and whole-genome sequence data. In summary, we suggested the polygenic inheritance of behavioral traits as well as the importance of the X-chromosome in the behavior modulation. Interestingly, about 50% and 10% of the candidate genes associated with livestock behavior were also statistically and causally associated with human diseases, respectively, where they were classified as behavioral and mental disorders, and nervous system diseases. Many pathways associated with sensory perception (e.g., olfaction), neuronal networks and brain functions (e.g., learning, adaptation, and memorization), and physiological mechanisms (e.g., steroids hormones) were identified. Furthermore, pathways related to both behavior and other livestock traits (e.g., meat quality) were reported. Key genes associated with behavioral traits were highlighted in this study, such as *NR3C2*, *RERG*, *PITPNM3*, *ZFAT*, *MAOB*, *OBP*, *HTR2C*, *MIR1912*, *U6*, *MAOA*, *OFD1*, *KCNK9*, *ZFAT*, and genes from olfactory and solute carrier families. The results of our study contribute to the understanding of behavior traits, independent of behavior assessments and environments to which the animals were defined subjectively. Additionally, we produced a list of candidate causal mutations in cattle that have significantly divergent allele frequencies between two breed groups with likely differential temperament characteristics (defined based on a subjective manner). Even though a solid conclusion cannot be drawn from these analyses alone due to well known within- and interbreed behavior variability, they provide auxiliary evidence supporting our findings. Those candidate genes and polymorphisms can be used as prior biological information to optimize genomic analyses (e.g., genome-wide association studies and genomic predictions) as well as describe polymorphisms to be added to existing genotyping platforms.

## Figures and Tables

**Table 1:** Breeds represented in the systematic review for the three species.

Species	Breed Background ^1^
**Cattle**	Angus, Angus Moiled, Australian Red, Blonde d’aquitaine, Brown Swiss, Brangus, Brahman, Belgian Blue, Charolais, Finnish Ayrshire, Guzerat, Gelbvieh, Hereford, Holstein, Jersey, Limousin, Montbeliarde, Normande, Red Angus, Simmental, Tarentaise
**Pigs**	Landrace, Large White, Duroc, White Duroc, Erhualian, Yorkshire, Meishan, Pietran, Songliao
**Sheep**	Merino, Romanov, Berrichon du Cher, Ram Mountain, Rideu-Arcott

^1^ The breeds may be represented by purebreds and/or crossbred animals.

**Table 2:** References, behavior-related trait, and number of genomic regions gathered for beef and dairy cattle.

Reference	Behavioral Trait (Number of Genomic Regions)	Reference	Behavioral Trait (Number of Genomic Regions)
Abo-Ismail et al. [58]	Milking speed (13) ^1^, Milking temperament (15)	Kolbehdari et al. [54]	Milking speed (20), Temperament (16)
Bailey et al. [34]	Terrain-use indices (rolling; 40), Terrain-use indices (rough; 42)	Kramer et al. [55]	Aggressiveness (2), Milking speed (5), Milking temperament (1), Rank order in herd (4), Temperament (2)
Boichard et al. [59]	Milking speed (4)	Lindholm-Perry et al. [49]	Flight speed (24)
Chan [52]	Temperament (21)	MacLeod et al. [56]	Temperament (10)
Santos [39]	Adjusted reactivity (14)	Marete et al. [38]	Milking speed (7)
Dreher et al. [71]	Sucking reflex (38)	Michenet et al. [30]	Maternal behavior (1)
Esmailizadeh et al. [93]	Blood cortisol level (1)	Sandor et al. [62]	Milking speed (2)
Friedrich et al. [94]	Duration activity (novel object test— OT; 12), Duration activity (open-field; 8), Duration inactivity (OT; 14), Duration inactivity (open-field; 18), Duration of the arena exploration (OT; 32), Duration of the arena exploration (open-field; 2)	Pierce [50]	Distance travelled from water (10), Elevation traversed (10), Percent slope traversed (10), Terrain-use indices (24)Terrain-use indices (rolling; 14), Terrain-use indices (rough; 14), Vertical climb (10)
Garza-Brenner [92]	Exit velocity (2), Pen score (4), Total score (Pen score and Exit velocity; 2)	Riley et al. [57]	Aggressive behavior (2), Temperament (6)
Garza-Brenner et al. [48]	Flight speed (4), Temperament (12)	Schmutz [40]	Behavior (6)
Glenske et al. [95]	Response to weighting process (2), Restraint docility test (1), Temperament (2)	Schrooten et al. [64]	Milking speed (2)
Guo et al. [69]	Milking speed (4)	Schrooten et al. [63]	Milking speed (9)
Gutierrez-Gil et al. [96]	Flight for feeder (6), Standing alert (3), Vocalization (22), Walking/running (9)	Spelman et al. [97]	Milking temperament (2)
Hanna et al. [53]	Temperament (66)	Valente et al. [33]	Flight speed (9)
Hiendleder et al. [37]	Milking speed (3)Temperament (3)	Valente et al. [98]	Daily feeding duration (3)Feeding event frequency (5)
Jardim et al. [61]	Milking speed (18)	Valle et al. [41]	Aggressive behavior (14), Locomotion (2), Maternal behavior (4)

^1^ The values in parentheses indicate the number of genomic regions reported.

**Table 3:** References, behavior-related trait, and number of genomic regions identified for pigs.

Reference	Behavioral Trait (Number of Genomic Regions)	Reference	Behavioral Trait (Number of Genomic Regions)
Bauer [65]	Infanticide (93) ^1^	Murani et al. [101]	Aggressive behavior (front lesions; 3; middle lesions; 2; rear lesions; 1; total lesions; 2), Cortisol level (1), Physiological stress (creatine kinase; 1), Physiological stress (glucose; 1)
Cross et al. [47]	Feeding behavior (66)	Murani et al. [102]	Cortisol level (27)
Desautes et al. [27]	Cortisol level (2), Cortisol level variations (1), Exploration during stress (1)	Ponsuksili et al. [46]	Backtest traits (latency, duration; and frequency of response; 132), Duration of struggling bouts (318), Frequency of struggling bouts (342), Latency at an age of 12 days (81)
Desire et al. [44]	Skin lesion traits (19)	Okamura et al. [103]	Cortisol level (1)
Ding et al. [104]	Mean feed intake/visit (1), Number of feeder visits/day (2), Time in the feeder/visit (1), Time in the feeder/day (2)	Quilter et al. [32]	Infanticide (8)
Do et al. [105]	Time in the feeder/day (12), Time in the feeder/visit (10)	Reiner et al. [106]	Time spent lying (1), Time spent socializing (1)
Gley et al. [107]	Coping behavior (20)	Reyer et al. [68]	Daily feeder visits (5), Daily feeding rate (8), Time in the feeder/day (9)
Gorres et al. [108]	Cortisol level (6)	Wurtz et al. [109]	Anterior lesions (1)
Guo et al. [69]	Daily feeding times (day 120 to 240; 1), Daily feeding times (day 210 to 240; 1), Daily visit times (day 210 to 240; 1), Feed intake/second (day 120 to 240; 2), Feed intake/second (day 210 to 240; 1), Feed intake/visit (day 120 to 240; 2)	Terenina et al. [99]	Adrenaline/creatinine level (2), Adrenaline/noradrenaline level (2) Cortisol/creatinine level (2), Cortisol+cortisone/creatinine level (3), Cortisone/cortisol ratios (2), Nor Adrenaline/creatinine level (2) Plasma cortisol at slaughter (3), Plasma creatine kinase at slaughter (1), Plasma glucose measured at slaughter (1), Plasma lactate at slaughter (1), Relative weight left adrenal gland (2), Relative weight right adrenal gland (2)
Hong et al. [43]	Social average daily gain (5)	Wilson et al. [67]	Tail biting (1), Victim of tail biting (5)
Houston et al. [110]	Time in the feeder/day (1)	Wu et al. [45]	Social genetic effect (average daily feed intake; 15), Social genetic effect (average daily gain; 19), Social genetic effect (backfat thickness to 100 kg; 17), Social genetic effect (backfat thickness; 25), Social genetic effect (days to 100 kg; 41), Social genetic effect (feed conversion ratio; 22), Social genetic effect (residual feed intake; 27)
Ma et al. [66]	Infanticide (1)		

^1^ The values in parentheses indicate the number of genomic regions reported.

**Table 4:** References, behavior-related trait, and number of genomic regions identified for sheep.

Reference	Behavioral Trait (Number of Genomic Regions)
Hazard et al. [35]	Distance from the human (corridor test 2; 4) ^1^, Locomotion (arena test 1; 2), Locomotion (arena test 2; 13), Locomotion (corridor test 1; 1), Locomotion (isolation box test; 2), Locomotion (social isolation; 2), The proximity to flock-mates and the human (arena test 2; 2), Time during which the human saw the lamb (corridor test 2; 1), Vocalization—high bleat (arena test 1; 14), Vocalization—high bleat (arena test 2; 10), Vocalization—high bleat (corridor test 1; 8), Vocalization—high bleat (isolation box test; 6), Vocalization—high bleat (social isolation; 26), Vocalization—low bleat (arena test 1; 18), Vocalization—low bleat (arena test 2; 7), Vocalization—low bleat (corridor test 1; 2), Vocalization—low bleat (social isolation; 10)
Pant et al. [111]	Endotoxin-induced cortisol response (16)
Poissant et al. [112]	Docility (1)
Qiu et al. [70]	Locomotion—frequency the animal crossed from one sector into another (1), Number of bleats (1)

^1^ The values in parentheses indicate the number of genomic regions reported.

**Table 5:** Selected candidate genes with their respective identification in the four species (i.e., cattle, human, pigs, and sheep) and chromosome (CHR) location. The selection of genes to present in this table depended on the recurrence of those genes within and across species as well as being reported in literature as biologically relevant genes affecting behavior.

Human Gene	Gene Description	Human CHR	Cattle Gene	Cattle CHR	Pig Gene	Pig CHR	Sheep Gene	Sheep CHR
*ADAM10*	ADAM metallopeptidase domain 10	15	*ADAM10*	10	*ADAM10*	1	*ENSOARG00000002399*	
*ADAMTS2*	ADAM metallopeptidase with thrombospondin type 1 motif 2	5	*ADAMTS2 ^1^*	7	*ADAMTS2*	2	*ADAMTS2*	5
*CAST*	Calpastatin	5	*CAST*	7	*CAST*	2	*CAST*	5
*CDC6*	Cell division cycle 6	17	*CDC6*	19	*CDC6*	12	*CDC6*	11
*CSE1L*	Chromosome segregation 1 like	20	*CSE1L*	13	*CSE1L*	17	*CSE1L*	13
*FAM135B*	Family with sequence similarity 135 member B	8	*FAM135B*	14	*FAM135B*	4	*FAM135B*	9
*FAM177A1*	Family with sequence similarity 177 member A1	14	*FAM177A1*	21	*FAM177A1*	7	*FAM177A1*	18
*GRM5*	Glutamate metabotropic receptor 5	11	*GRM5*	29	*GRM5*	9	*ENSOARG00000014407*	21
*HTR2C*	5-Hydroxytryptamine receptor 2C	X	*HTR2C*	X	*HTR2C*	X	*HTR2C*	X
*KCNK9*	Potassium two pore domain channel subfamily K member 9	8	*KCNK9*	14	*KCNK9*	4	*ENSOARG00000004303*	9
*MACROD2*	Mono-ADP ribosylhydrolase 2	20	*ENSBTAG00000048850*	13	*MACROD2*	17	*ENSOARG00000011613*	13
*MAML3*	Mastermind like transcriptional coactivator 3	4	*MAML3*	17	*MAML3*	8	*MAML3*	17
*MAOA*	Monoamine oxidase A	X	*MAOA*	X	*ENSSSCG00000012257*	X	*MAOA*	X
*MAOB*	Monoamine oxidase B	X	*MAOB*	X	*MAOB*	X	*MAOB*	X
*MIR1912*	MicroRNA 1912	X			*MIR1912*	X	*MIR1912*	X
*MUSK*	Muscle associated receptor tyrosine kinase	9	*MUSK*	8	*MUSK*	1	*MUSK*	2
*NR3C2*	Nuclear receptor subfamily 3 group C member 2	4	*NR3C2*	17	*NR3C2*	8	*ENSOARG00000007116*	17
*OFD1*	OFD1 centriole and centriolar satellite protein	X	*OFD1*	X	*ENSSSCG00000012125*	X	*OFD1*	X
*OR2B6*	Olfactory receptor family 2 subfamily B member 6	6	*OR2B6*	23	*OR2B6*	7	*ENSOARG00000009162*	20
*OR51E2*	Olfactory receptor family 51 subfamily E member 2	11	*OR51E2*	15	*OR51E2*	9	*OR51E2*	15
*OR52J3*	Olfactory receptor family 52 subfamily J member 3	11	*ENSBTAG00000038075*	15	*OR52J3*	9	*ENSOARG00000006229*	15
*OR56A1*	Olfactory receptor family 56 subfamily A member 1	11	*OR56A1*	15	*OR56A1*	9	*OR56A1*	15
*OR9Q2*	Olfactory receptor family 9 subfamily Q member 2	11	*OR9Q2*	15	*OR9Q2*	2	*OR9Q2*	15
*PITPNM3*	PITPNM family member 3	17	*PITPNM3*	19	*PITPNM3*	12	*PITPNM3*	11
*PLCB1*	Phospholipase C beta 1	20	*PLCB1*	13	*PLCB1*	17	*PLCB1*	13
*POMC*	Proopiomelanocortin	2	*POMC*	11	*ENSSSCG00000033439*		*POMC*	3
*RERG*	RAS like estrogen regulated growth inhibitor	12	*RERG*	5	*RERG*	5	*RERG*	3
*SLC13A5*	Solute carrier family 13 member 5	17	*SLC13A5*	19	*SLC13A5*	12	*SLC13A5*	11
*SLC16A11*	Solute carrier family 16 member 11	17	*SLC16A11*	19	*SLC16A11*	12	*SLC16A11*	11
*SLC18A2*	Solute carrier family 18 member A2	10	*SLC18A2*	26	*SLC18A2*	14	*SLC18A2*	22
*SLC25A4*	Solute carrier family 25 member 4	4	*SLC25A4*	27	*SLC25A4*	15	*SLC25A4*	26
*SLC5A2*	Solute carrier family 5 member 2	16	*SLC5A2*	25	*SLC5A2*	3	*SLC5A2*	24
*SLC6A2*	Solute carrier family 6 member 2	16	*SLC6A2*	18	*SLC6A2*	6	*SLC6A2*	14
*SPNS3*	Sphingolipid transporter 3 (putative)	17	*SPNS3*	19	*SPNS3*	12	*SPNS3*	11
*ZFAT*	Zinc finger and AT-hook domain containing	8	*ZFAT*	14	*ZFAT*	4	*ZFAT*	9

CHR: chromosome; *^1^* Bold print genes represent the species in which the gene was found to be associated with behavior trait.

## Data Availability

All data used in this study are included in the main text and Supplementary Material (Tables S1A–C).

## Supplementary Materials

The following are available online at https://www.mdpi.com/2076-2615/11/3/715/s1, Figure S1. The geographical distribution of the data gathered in this systematic review. The letter indicates the species: (A) beef and dairy cattle, (B) pigs, and (C) sheep. The color represents the number of genomic regions reported to be associated with behavior-related traits per country: gold—from one to 10, blue—from 11 to 50, orange—from 51 to 100, red—from 101 to 200, and green—more than 201 genomic regions associated with behavioral traits. The labelled boxes in the right are the breeds represented per country. Table S1. Genomic regions associated with behavioral traits gathered in the present study. The letter (worksheet) indicates the species: (A) beef and dairy cattle, (B) pigs, and (C) sheep. Publication information (i.e., DOI), behavioral trait measured, genomic region coordinates (i.e., chromosome, start and end position), genome assembly, marker name, flank gene, and description of the dataset used (e.g., breed, country, number of markers, number of the individuals, number of genotyped individuals) and analysis performed (e.g., analysis type, fixed and random effects accounted in the model, software, heritability) are presented (XLSX). Table S2. Candidate genes identified in farmed mammals. The letter (worksheet) indicates the species: (A) beef and dairy cattle (genome assembly ARS-UCD1.2), (B) pigs (genome assembly Sscrofa11.1), and (C) sheep (genome assembly OAR 3.0). The Ensembl gene identification, external gene name, and genome coordinate (i.e., chromosome, start and end position) are presented. Additionally, homologous gene information in other farmed mammals (i.e., cattle, pigs, and sheep) and human were provided per species (XLSX). Table S3. Recurrence of candidate genes among genomic regions annotated in farmed mammals. The letter (worksheet) indicates the species: (A) beef and dairy cattle (genome assembly ARS-UCD1.2), (B) pigs (genome assembly Sscrofa11.1), and (C) sheep (genome assembly OAR 3.0). Each worksheet shows the gene identification (i.e., gene name and Ensembl identification), genome coordinate, frequency of gene appearance (how many times the gene appeared within the genomic regions gathered), reference and trait in which the gene were identified (XLSX). Table S4. DAVID functional annotation analysis performed for cattle. The letters indicate the functional enrichment category: (A) Gene Ontology biological process terms, (B) Gene Ontology cellular component terms, (C) Gene Ontology molecular function terms, (D) Kyoto Encyclopedia of Genes and Genomes (KEGG), and (E) Functional clustering (XLSX). Table S5. Significantly different polymorphisms within exon regions of cattle candidate genes using contrasting temperament breeds and whole-genome sequence. The letters indicate the subset of loci and genes. (A) All significantly different loci by contrasting more docile and more aggressive breed groups. (B) Significant differed loci considering a power of Fisher’s exact test higher than 80% (XLSX). Table S6. DAVID functional annotation analysis performed for pigs. The letters indicate the functional enrichment category: (A) Gene Ontology biological process terms, (B) Gene Ontology cellular component terms (C) Gene Ontology molecular function terms, and (D) Kyoto Encyclopedia of Genes and Genomes (KEGG) (XLSX). Table S7. DAVID functional annotation analysis performed for combined gene-set. The letters indicate the functional enrichment category: (A) Gene Ontology biological process terms, (B) Gene Ontology cellular component terms, (C) Gene Ontology molecular function terms, and (D) Kyoto Encyclopedia of Genes and Genomes (KEGG) (XLSX). Table S8. Key candidate genes statistically associated with human diseases (behavioral, mental, and neuronal disorders) (XLSX). Table S9. Key candidate genes reported in causal association studies for human diseases (behavioral, mental, and neuronal disorders) (XLSX). Table S10. Significantly different polymorphisms within exon regions of combined candidate gene-set using contrasting temperament breeds and whole-genome sequence. (A) All significantly different loci by contrasting more docile and more aggressive breed groups. (B) Significant differed loci considering a power of Fisher’s exact test higher than 80% (XLSX).
