# Supplementary material for: A Systematic Review of Genomic Regions and Candidate Genes Underlying Behavioral Traits in Farmed Mammals and Their Link with Human Disorders
Source: Animals (Basel). 2021 Mar 6;11(3):715. doi: 10.3390/ani11030715 (PMC7999279; doi:10.3390/ani11030715)
Supplement: Supplementary file 1 [file animals-11-00715-s001.zip › 2.SupplementaryMaterial/1.Supplementary figures.docx]

**Supplementary figures**

**
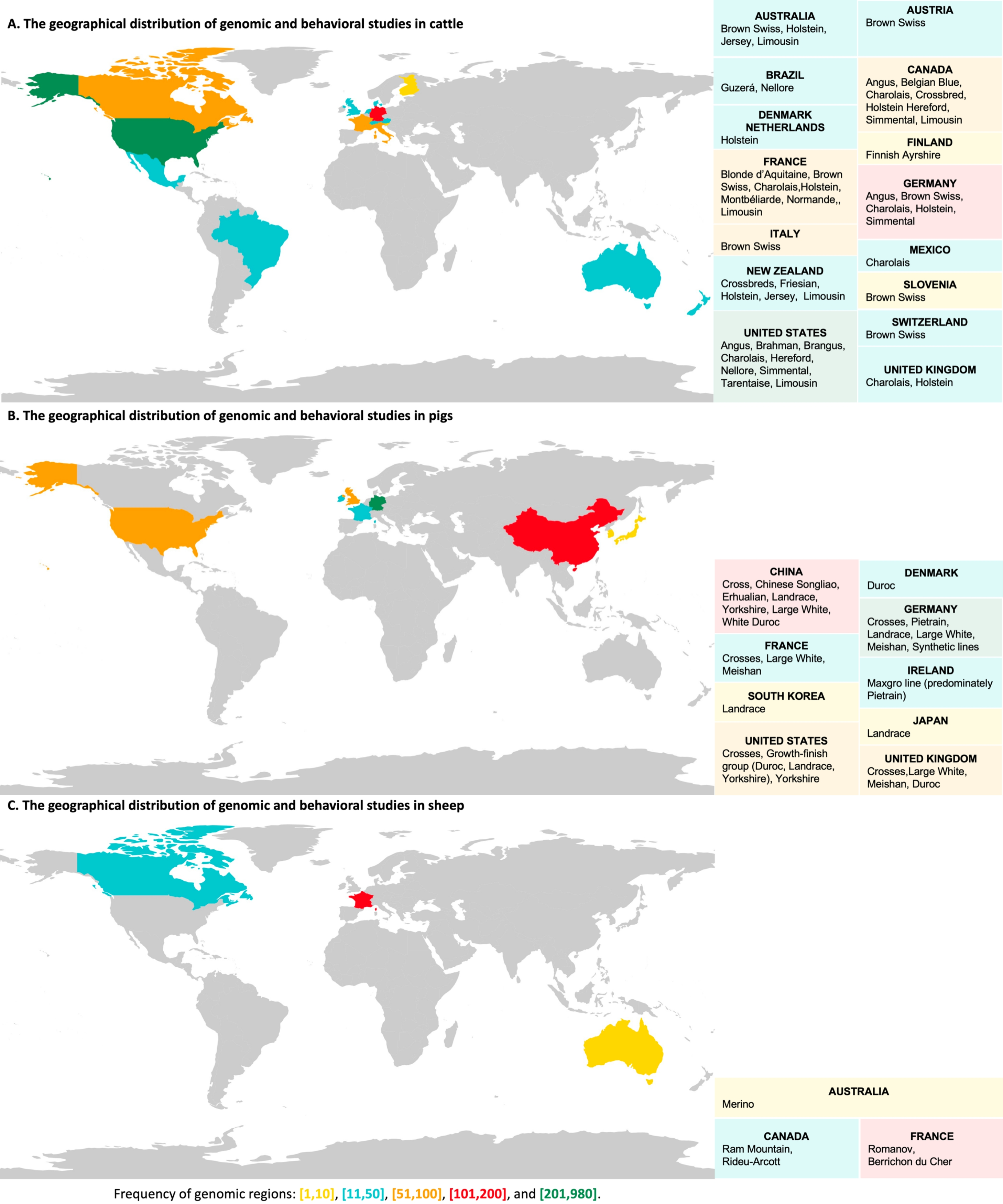
**

**Figure S1.** The geographical distribution of the data gathered in this systematic review. The letter indicates the species: (A) beef and dairy cattle, (B) pigs, and (C) sheep. The color represents the number of genomic regions reported to be associated with behavior-related traits per country: gold—from one to 10, blue—from 11 to 50, orange—from 51 to 100, red—from 101 to 200, and green—more than 201 genomic regions associated with be-havioral traits. The labelled boxes in the right are the breeds represented per country.
